# Functional responses of key marine bacteria to environmental change – toward genetic counselling for coastal waters

**DOI:** 10.3389/fmicb.2022.869093

**Published:** 2022-12-01

**Authors:** Jarone Pinhassi, Hanna Farnelid, Sandra Martínez García, Eva Teira, Pierre E. Galand, Ingrid Obernosterer, Christopher Quince, Maria Vila-Costa, Josep M. Gasol, Daniel Lundin, Anders F. Andersson, Matthias Labrenz, Lasse Riemann

**Affiliations:** ^1^Centre for Ecology and Evolution in Microbial Model Systems, Linnaeus University, Kalmar, Sweden; ^2^Departamento de Ecoloxía e Bioloxía Animal, Centro de Investigación Mariña, Universidade de Vigo, Vigo, Spain; ^3^CNRS, Laboratoire d’Ecogéochimie des Environnements Benthiques (LECOB), Sorbonne Université, Banyuls-sur-Mer, France; ^4^CNRS, Laboratoire d’Océanographie Microbienne (LOMIC), Sorbonne Université, Banyuls-sur-Mer, France; ^5^Earlham Institute, Norwich, United Kingdom; ^6^IDAEA-CSIC, Barcelona, Spain; ^7^Institut de Ciències del Mar-CSIC, Barcelona, Spain; ^8^Department of Gene Technology, Science for Life Laboratory, KTH Royal Institute of Technology, Stockholm, Sweden; ^9^Leibniz-Institute for Baltic Sea Research, Rostock, Germany; ^10^Marine Biology Section, Department of Biology, University of Copenhagen, Helsingør, Denmark

**Keywords:** marine bacteria, ecosystem service, water quality, management, traits

## Abstract

Coastal ecosystems deteriorate globally due to human-induced stress factors, like nutrient loading and pollution. Bacteria are critical to marine ecosystems, e.g., by regulating nutrient cycles, synthesizing vitamins, or degrading pollutants, thereby providing essential ecosystem services ultimately affecting economic activities. Yet, until now bacteria are overlooked both as mediators and indicators of ecosystem health, mainly due to methodological limitations in assessing bacterial ecosystem functions. However, these limitations are largely overcome by the advances in molecular biology and bioinformatics methods for characterizing the genetics that underlie functional traits of key bacterial populations – “key” in providing important ecosystem services, being abundant, or by possessing high metabolic rates. It is therefore timely to analyze and define the functional responses of bacteria to human-induced effects on coastal ecosystem health. We posit that categorizing the responses of key marine bacterial populations to changes in environmental conditions through modern microbial oceanography methods will allow establishing the nascent field of genetic counselling for our coastal waters. This requires systematic field studies of linkages between functional traits of key bacterial populations and their ecosystem functions in coastal seas, complemented with systematic experimental analyses of the responses to different stressors. Research and training in environmental management along with dissemination of results and dialogue with societal actors are equally important to ensure the role of bacteria is understood as fundamentally important for coastal ecosystems. Using the responses of microorganisms as a tool to develop genetic counselling for coastal ecosystems can ultimately allow for integrating bacteria as indicators of environmental change.

## Introduction

Earth is under tremendous pressure from human activities and the resulting global change. Coastal waters are productive ecosystems of high importance to society that suffer greatly from human activities causing water pollution, eutrophication, disrupted nutrient cycles, and loss of biodiversity (Rockström et al., 2009; Nash et al., 2017; Halpern et al., 2019). Legislative regulations for coastal management differ substantially between countries and continents, which represents a challenge for international agreements to promote coastal ecosystem health. As an example from the continent where we have our affiliations, it can be noted that the overarching aim of the Marine Strategy Framework Directive developed by the European Commission is to attain good environmental status across Europe’s marine environment. We think that achieving good environmental status in coastal marine waters requires actions based on due knowledge of the major ecosystem components – and an essential organism group that is left out of the equation is the bacteria (here denoting prokaryotes; Bacteria and Archaea).

**Figure 1:**
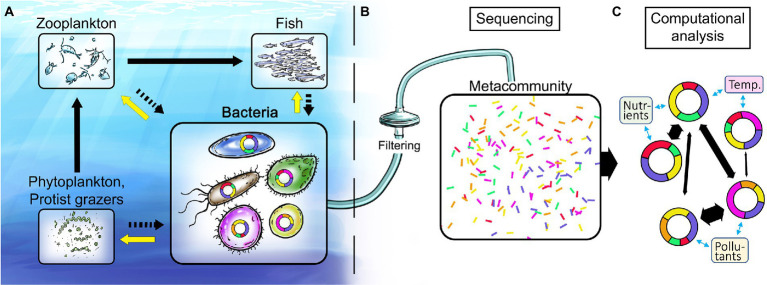
Illustration of the marine food web and proposed analysis workflow for disentangling linkages between environmental stressors and genomic structure and content of key marine bacteria. **(A)** Bacteria are fundamental in recycling of nutrients including dissolved organic carbon (black arrows to bacteria) and in providing ecosystem-wide services to higher trophic levels (yellow arrows). **(B)** Current state of the art methodology is based on sampling of the total nucleic acid (DNA and/or RNA) content of marine environments. **(C)** Computational analyses provide metagenome-assembled genomes (MAGs) that give unprecedented insights into both the identity of key bacterial populations and the genes underlying their different functional traits. Doughnut shapes represent genomes of such key bacterial populations and the colored doughnut sections denote traits. Co-occurrence network analysis of bacterial populations and their traits (black arrows between genomes) allows for identification of traits responsive to global change variables (blue arrows) and ultimately the ecosystem services supplied by key bacteria.

So, why consider bacteria? Following the ground-breaking discovery in the early 1980s that planktonic bacteria in the oceans are actively growing and extremely abundant (10^9^ cells/L), blossoming research on the metabolism, biodiversity and ecology of marine bacteria rapidly established their paramount role in the degradation of dissolved organic carbon (DOC). Moreover, bacteria regulate biogeochemical element cycles and influence overall marine productivity, processing more than 50% of the carbon fixed by photosynthesis and mediating most transformations of, e.g., nitrogen, phosphorus, and trace metals (Cole et al., 1988; Azam, 1998; Falkowski et al., 2008; Figure 1A). Recent advances in microbial oceanography have revealed that bacteria also provide a number of unexpected goods and services that influence ecosystem dynamics and productivity up to the level of fish, including the degradation of pollutants, production of vitamins, degradation of reactive oxygen species and the production of growth hormones (Amin et al., 2015; Durham et al., 2015; Cavicchioli et al., 2019). Moreover, because of their fast growth rates, bacteria react sensitively and rapidly to changes in environmental conditions. Nevertheless, bacteria have so far essentially been overlooked as indicators of ecosystem health, which has recently been highlighted (Zhang et al., 2020; Cordier et al., 2021; Alonso et al., 2022; Orel et al., 2022), and are currently not integrated in the development of policy making and management strategies to meet and mitigate the challenges imposed by human activity and global change on coastal waters.

An analogy between human health and ecosystem health is warranted to grasp the potential of the mapping of the genetic basis for bacterial responses to environmental change. In medical practice, obtaining information about genetic disorders to make individually informed choices on medication and risk management – based on advice from genetics counsellors and medical doctors – is a rapidly spreading practice at hospitals worldwide, thanks to the large amount of affordable information gained by genome sequencing of patient material (be it cancer cells or gut microbiomes). In medicine, knowledge sources ranging from patient records to results from animal disease-model experiments (just to mention a few) have been essential to develop the understanding necessary to develop efficient treatments. In marine research, knowledge from time series measurements in the sea and controlled experiments with bacterial assemblages and isolates constitute a corresponding knowledge base (McCarren et al., 2010; Palovaara et al., 2014; Fuhrman et al., 2015; Bunse and Pinhassi, 2017; Landa et al., 2017). We find that it is now feasible and pertinent to build the knowledge for genetic counselling for the environment – effectively to interpret and predict alterations in microbial ecosystem functions in response to global change (Figure 1).

This is the background against which we call for systematically deploying state-of-the-art methods for genetic and genomic analyses of coastal ecosystems. This would permit identifying and characterizing functional traits of key bacterial populations, so that ultimately – through a dialogue between researchers and policy makers – bacteria can be implemented in environmental monitoring and used to improve the management of our world’s coastal waters.

### Interrogating linkages between bacterial traits and ecosystem functions

Why are bacteria not yet explicitly considered in policy making, marine management practices, or ecosystem models? One central explanation is the lack of morphological traits that distinguish components of marine bacterioplankton – e.g. families, species or populations – which in turn severely complicates the study of their ecology. In this context, it is enlightening to make a comparison with another central plankton group, the photosynthetic eukaryotic and prokaryotic microorganisms referred to as phytoplankton. Importantly, the pronounced (and captivating) morphological differences between several phytoplankton groups allowed aquatic ecologists already in the early 1900s to establish a thorough understanding of functional traits of key taxa in a quantitative manner. Further, the spatiotemporal distribution of key phytoplankton taxa could be shown to vary predictably in relation to environmental conditions (Falkowski and Oliver, 2007). Therefore, major phytoplankton taxa, differing for example in size, motility or pigmentation, are represented in contemporary ecosystem models used for providing guidelines on sustainable management practices of aquatic environments around the globe (e.g., the BALTSEM model for the Baltic Sea). Conceptual and methodological advances in microbial ecology now allow for genetic and genomic characterization of the molecular mechanisms that determine the ecological niches of bacterial taxa (Figures 1B,C). We therefore posit that it is now both feasible and pertinent to develop corresponding knowledge of the metabolic and physiological characteristics representing functional traits that distinguish key bacterial taxa (Martiny et al., 2015; Coles et al., 2017; Baltar et al., 2019). Identification of such functional traits will be the first step to allow for a future inclusion of bacteria in ecosystem models.

### Bacterial traits and ecosystem function

To obtain a thorough and mechanistic understanding of ecosystem functioning we reason that it is important to maneuver aquatic microbial ecology into the territory of trait-based ecology (Raes et al., 2011; Wallenstein and Hall, 2012; Brown et al., 2014; Krause et al., 2014). This includes defining what traits are and how they can be understood. One such example relates to microbial vitamin metabolism. The entire traditional food chain up to fish and birds depends on vitamin B1 produced by microbes. For this, it is possible to determine which bacteria contain the genes encoding vitamin B1 synthesis pathways and assign them to the trait “vitamin B1 synthesis,” and thus identifying the main providers of the ecosystem service “vitamin provision” (Paerl et al., 2018). In a similar manner, one can explore the role of bacteria in carbon cycling through analysis of traits like polysaccharide metabolism (conferred by, e.g., glycosyl hydrolases) and hydrocarbon metabolism (conferred by, e.g., aromatic-ring- hydroxylating dioxygenase genes; this at the same time addresses pollutant degradation traits (González-Gaya et al., 2019)). Also a variety of nitrogen and phosphorus cycling-related traits are pertinent to characterize, such as those for uptake or metabolism of different classes of dissolved organic or inorganic nitrogen or phosphorus. This could include genes for aminopeptidases versus phosphatases, which cross talk with the carbon cycle, or more specific traits like polyphosphate metabolism (conferred by purine nucleoside phosphorylases, PNPs), and genes involved in nitrification, denitrification or nitrogen fixation (Happel et al., 2019; Sosa et al., 2019). We recognize the challenge associated with defining traits, at an appropriately specific level, to obtain ecologically meaningful interpretations of patterns of trait distribution in nature. Nevertheless, we find that even traits carried by diverse taxa, e.g., nitrogen fixation (Zehr et al., 2003; Hallstrøm et al., 2022), provide pivotal insights into the interactions between microbes and the surrounding environment.

### The potential to characterize linkages between bacteria and ecosystem services

A primary requisite to identify the genetics underlying particular traits is the access to detailed data on the genomes of key bacterial populations, which circumvents the dependence on difficult-to-assess differences in morphological or other phenotypic distinctions between taxa. In fact, standards are being developed so that new taxa and their names can be proposed based entirely on genomic information (Hedlund et al., 2022). Due to increasingly high-throughput and cost-effective sequencing methodologies, knowledge is accumulating on the spatiotemporal distribution of microbial groups and populations. In recent years, amplicon sequencing of phylogenetic marker genes (e.g., 16S rRNA) has been complemented by metagenomic and metatranscriptomic (−omics) studies of natural marine communities. Long-term field sampling provides important knowledge on the seasonal dynamics of key bacterial populations (Fuhrman et al., 2006; Teeling et al., 2012; Cram et al., 2015; Lambert et al., 2019, 2021; Auladell et al., 2022), and it has become evident that several ecosystem services are directly dependent on the diversity of bacterial communities (Galand et al., 2015, 2018; Garcia-Garcia et al., 2019). This complements theoretical considerations showing that biodiversity is fundamental for the capacity of natural communities to adapt to environmental change, i.e., the insurance effects of biodiversity (Yachi and Loreau, 1999). However, there is a critical lack of knowledge as to *why* different bacterial populations become dominant at different times and places or, even more importantly, *how* different populations influence their environment (i.e., what ecosystem functions they carry out). The answers to these questions are now tractable through molecular studies of the regulation of functional traits encoded in the genomes of key bacterial populations in response to environmental factors (Krause et al., 2014). Hereby we emphasize the use of -omics analyses in combination with the study of physiological and ecological responses. Moreover, the -omics approaches have the potential to resolve evolutionary long term adjustments in microbial communities.

The potential of linking -omics and environmental/ecological data has only recently been explored (Garcia et al., 2020; VanInsberghe et al., 2020), in part due to a lack of adequate computational methods (bioinformatics). Pioneering advances forming the basis for genetic counselling for the environment span from: 1) mapping bacterial taxa and their dynamics through 16S rRNA gene amplicon sequencing (Andersson et al., 2010; Edgar et al., 2011; Herlemann et al., 2011), *via* 2) the development of *in situ* instrumentation as a basis for non-biased -omics analyses (Ottesen et al., 2011; Bochdansky et al., 2017; Charvet et al., 2019), and 3) metagenomics and metatranscriptomics whereby functional genes are identified and their expression quantified (Gifford et al., 2013; Bunse et al., 2016; Rognes et al., 2016; Markussen et al., 2018; Alneberg et al., 2018b; Oberbeckmann et al., 2021), to 4) the present frontier, where it is possible to define bacterial populations and obtain genomes for key populations from metagenomic datasets, i.e., metagenome-assembled genomes (MAGs; Albertsen et al., 2013; Alneberg et al., 2014; Hugerth et al., 2015; Alneberg et al., 2018a; Figure 1C **)**. Moreover, from the content of functional genes in MAGs, their ecological niches across gradients of environmental conditions can be predicted, e.g., temperature, salinity, (in) organic nutrients and pollutants (Delmont et al., 2019; Alneberg et al., 2020; Cerro-Galvez et al., 2021; Pereira et al., 2021; Sjöqvist et al., 2021; Sun et al., 2021). Ultimately, this will move the field forward by determining how ecosystem services are distributed among key bacterial populations, rather than at community level as done before.

Adoption of MAG-assembly combined with long read sequencing approaches have recently given insights into the distribution of bacterial populations and their traits across the vastness of the open ocean (Ibarbalz et al., 2019), while leaving major blank spaces for the coastal seas. MAGs combined with metagenomic data sampled across environmental gradients can even reveal intra-species population structures and genes under environment-specific selection (Delmont et al., 2019; Sjöqvist et al., 2021). The finding of such strong links between bacterial genomes and the ecological niche provides a solid conceptual framework for predictive ecology based on genomic data from the environment. This allows, for the first time, to resolve some long standing yet urgent tasks: to determine how ecosystem services are distributed among marine bacteria (Ducklow, 2008), how key bacterial populations are affected by human impacts on coastal waters (Hutchins and Fu, 2017), and, in turn, how changes in bacterial abundances and activities influence the ecosystem, and vice versa (Azam and Malfatti, 2007). Now it is time to apply this knowledge to assess how human pressures on coastal waters influence bacterial communities and their ecosystem services. This has the potential to contribute evidence-based knowledge in both academic and non-academic settings where policies are shaped.

### Research strategy into the future

We propose that systematic study of the microbial oceanography in coastal seas through the lenses of molecular biosciences could pave the way for the integration of bacteria into environmental management. In practice, we envision the following “road map”:

1. Compilation of prokaryotic biodiversity data (16S rRNA gene amplicons) from coastal locations, i.e., compilation and meta-analysis of existing data sets by taking advantage of existing projects and tools.
2. Establishment of MAG libraries from metagenomes across coastal locations of interest; through field sampling to identify key bacterial populations and their functional potentials; i.e. putative traits.
3. Identification of expressed traits from metatranscriptomes; field study catalogue of expressed genes from prokaryotic communities in general, and key bacterial populations (MAGs) in particular, from coastal locations.
4. Experimental testing of the regulation of key traits; environmental factors and anthropogenic stressors as determinants of trait responses of bacterial assemblages and key bacterial populations (MAGs).
5. Experimental testing of anthropogenic stressor effects on functional responses and traits in single isolates or mixtures of isolates representative of the major taxa of coastal marine bacterial communities.
6. Cross validation of trait responses to individual stressors in key bacterial populations of different coastal seas (international meta-analysis of linkages between environmental stressors and particular bacterial functional traits and taxa).
7. Establishment of national or regional “meeting points” for dialogue and collaborations with society – national environmental protection agencies, and local and regional authorities and industry.
8. Establishment of an international collaborative group to generate efficient layman descriptions/summaries of research findings – aimed at general public/education and authorities – to outline the utility of genetic counselling for the environment.

**Figure 2:**
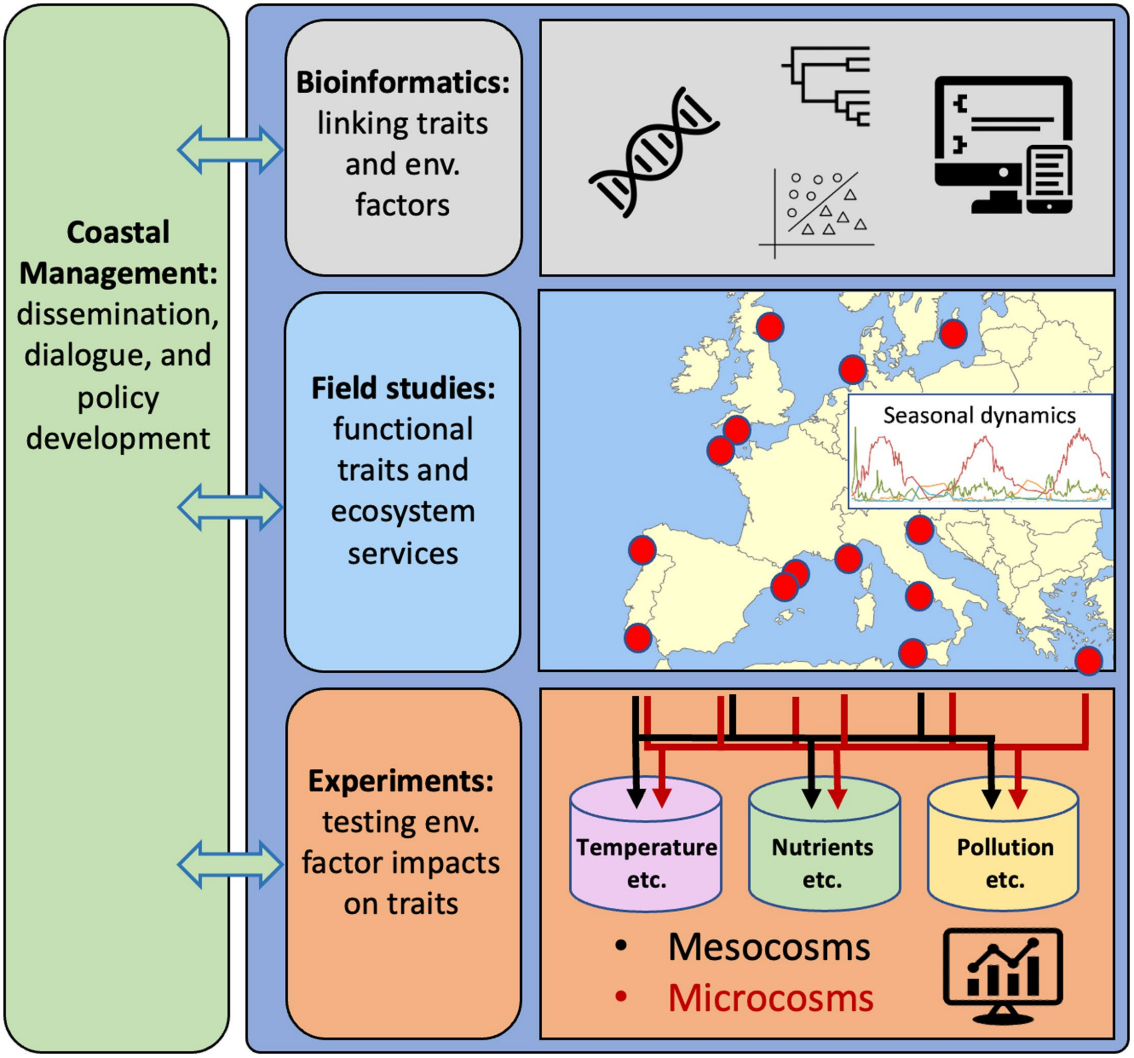
Research strategy to obtain comprehensive understanding of the genetic responses of key marine bacteria to environmental stressors in coastal waters, using European waters as an example. Bioinformatics for developing computational tools and workflows to effectively interpret linkages between functional traits and environmental factors. Field studies to generate and evaluate data on traits of key bacterial populations (from genomes and transcriptomes) and their distributions in time and space in relation to variability in environmental conditions. Experiments to identify and confirm relationships between environmental factors and specific traits and bacterial populations. Synergies between computational analyses of genomics, distributions and measured variability in environmental variables from field studies and experiments are essential to identify how bacterial functional traits can form the basis for genetic counselling of coastal seas. Dialogs with societal actors through all steps in the research strategy will be needed for reaching the goal of using bacteria to improve the quality of actions in coastal management.

We think that systematically following such a road map would decipher the functionality and regulation of key bacterial populations in coastal waters. To reach this goal, it would be necessary to consolidate and develop existing computational methods for linking genes and populations to ecosystem services (Figures 1B,C, 2, “Bioinformatics”). An ambitious goal is then to delineate and ultimately predict the functionality of key bacterioplankton organisms *via* approaches ranging in complexity from time-resolved *in situ* studies (Figure 2, “Field studies”) to hypothesis-driven experiments with natural assemblages and/or experiments with single and multiple bacterial strains (Figure 2, “Experiments”). Such experiments could be done in both microcosms (up to some liters) and mesocosms (hundreds of liters) to address scale-dependent ecological processes. Together, these approaches are suited to address the overarching aim of resolving how traits and ecosystem functions are distributed among key bacterial populations present in coastal ecosystems, and how the abundance and activity of these bacteria – and thus their ecological traits – are affected by environmental factors in general, and environmental factors directly affected by global change in particular. In this framework, marine stations along the coasts of Europe are used as an example (Figure 2), but it can be applied and implemented globally. In parallel with these advances, it is necessary to engage in outreach and dissemination of research findings to a broad public along with training to bridge knowledge of microbial ecology and microbiome research with understanding of practices in management and governance (Figure 2, “Coastal Management”).

## Discussion

A fundamental incentive for this Perspective is the unifying framework of molecular biosciences, which daily brings important new knowledge to humanity, be it on human evolution, history, forensics, or medical sciences. Microbial oceanography pioneered the study of bacterial ecology and diversity using molecular methods (Giovannoni et al., 1990; Fuhrman et al., 1992; Béjà et al., 2000; Venter et al., 2004), setting the stage for the ongoing quest in environmental and medical sciences alike, to understand the interdependencies between microbiomes (i.e., microbial community composition and dynamics) and their “environment,” using the “omics” approaches (genomics, transcriptomics and proteomics; applied to tissues, cell lines, model bacteria and natural communities [termed “meta-omics” when applied to the latter]).

Coastal waters are highly valuable ecosystems with regard to the services they provide; e.g. fish and shellfish production, recreation, and waste assimilation. Understanding the factors that influence the productivity and stability of coastal ecosystems is therefore of utmost importance for a sustainable management of these regions. Working toward implementing genetic counselling for the marine environment is pertinent because increasing anthropogenically induced environmental disturbances, such as eutrophication and climate change, are placing numerous stressors on life in the oceans. It is also timely because, for the first time, we can decipher the functional role of key bacterial populations. *Via* advanced molecular techniques, the genes maintained and expressed by microbes can now be identified – revealing the functionality of these organisms and their linkages to prevailing environmental conditions and nutrient biogeochemistry. This facilitates the prediction of ecosystem services provided by global coastal waters in the future – a prerequisite for early mitigation of human impact and a critical step for identifying the societal actions required to ensure sustainable management of these sensitive regions in the face of global change.

We think it is timely to incorporate the emerging knowledge of genetics and ecology of bacteria – understood as genetics counselling for the environment – into developing increasingly sustainable management strategies and evaluation processes (Cordier et al., 2021). Beyond the systematic exploration of the relation between functional traits of key bacterial populations and environmental conditions, this requires education of the next generation of researchers with special emphasis on bridging bioinformatics, molecular biology, marine ecology, and ecosystem management. A prerequisite for success in this direction is interdisciplinary research involving modern environmental microbiology – computational and molecular biology, physiology, microbial oceanography and ecology – along with involvement of stakeholders in coastal management. Dissemination of results is an important component, as nicely exemplified through the TARA oceans project.^1^

In the outlined research strategy, continued use of and design of new coastal time-series stations will be critical for cross-system identification of environmental drivers of bacterial functionality – linkages between the environment and the processes carried out by bacteria. Time-series meta-omics datasets of at least 5–10 years are important because nutrient levels and biological activities are dynamic and seasonal features of temperate coastal ecosystems are expected to be affected by climate change. In turn, the established links furnish an ecophysiological understanding of key bacterial populations in natural waters, and this will be a precondition for the successful accomplishment of points 6 to 8 above. Research communication and dialogue with society has recently been emphasized in policy documents from, e.g., UNESCO, Global Research Council and Science Europe. Such closer collaboration between researchers and societal actors can be partially supported by national funding sources, but for example the efforts to reach a shared understanding across borders (emphasized in the last three points) would benefit from international funding. Certainly, researcher engagement in defining future (inter)national funding schemes would be warranted for this. The proposed actions have the potential to ensure that the role of bacteria is recognized as being of fundamental importance for the coastal marine ecosystem, and that this knowledge reaches decision makers involved in marine management. This will be invaluable for the continued refinement of sustainable management strategies for coastal zones.

## Conclusion

We envision that microbes are sensitive sentinels (allowing detection of for example pathogens, nutrient loadings, pollution, and also in the long run as indicators of changes in food web structure in response to, e.g., over fishing) for the identification of undesired changes in the environment. With this view, the use of coastal microorganisms in genetic counselling for the environment could provide guidance for actions to reduce anthropogenic impact on the environment, which involves, e.g., the creation of marine reserves/protected areas or actual changes in legislation for the use of, e.g., fertilizers and pesticides. We posit that the actions presented in our road map for systematically exploring the responsiveness of key bacterial populations to environmental change – in time and space – will become critically important for laying the foundations for future assessment of environmental quality, optimization of coastal management, and policy. This is an urgent issue given the very rapid conceptual and methodological developments in using genetic and genomic analyses to obtain mechanistic understanding of ecological processes – paralleling and interacting with the corresponding developments in, e.g., medical sciences, forensics, biotechnology, and other industries. We believe that informed decisions based on environmental genetic counselling will ultimately aid mitigation of threats to coastal ecosystems posed by human activity, such as global change.

## Data availability statement

The original contributions presented in the study are included in the article/supplementary material, further inquiries can be directed to the corresponding author.

## Author contributions

All authors listed have made a substantial, direct, and intellectual contribution to the work and approved it for publication.

## Conflict of interest

The authors declare that the research was conducted in the absence of any commercial or financial relationships that could be construed as a potential conflict of interest.

## Publisher’s note

All claims expressed in this article are solely those of the authors and do not necessarily represent those of their affiliated organizations, or those of the publisher, the editors and the reviewers. Any product that may be evaluated in this article, or claim that may be made by its manufacturer, is not guaranteed or endorsed by the publisher.

## Footnotes

^1^ https://www.embl.org/topics/tara/
